# CD8+ T Cell Co-Expressed Genes Correlate With Clinical Phenotype and Microenvironments of Urothelial Cancer

**DOI:** 10.3389/fonc.2020.553399

**Published:** 2020-11-19

**Authors:** Yutao Wang, Kexin Yan, Jiaxing Lin, Yang Liu, Jianfeng Wang, Xuejie Li, Xinxin Li, Zhixiong Hua, Zhenhua Zheng, Jianxiu Shi, Siqing Sun, Jianbin Bi

**Affiliations:** ^1^ Department of Urology, China Medical University, The First Hospital of China Medical University, Shenyang, China; ^2^ Department of Dermatology, China Medical University, The First Hospital of China Medical University, Shenyang, China

**Keywords:** ****CD8^+^ T cells, antigen presentation, weighted gene co-expression network analysis, immune microenvironment, bladder cancer

## Abstract

**Purpose:**

To identify immune-related co-expressed genes that promote CD8^+^ T cell infiltration in bladder cancer, and to explore the interactions among relevant genes in the tumor microenvironment.

**Method:**

We obtained bladder cancer gene matrix and clinical information data from TCGA, GSE32894 and GSE48075. The “estimate” package was used to calculate tumor purity and immune score. The CIBERSORT algorithm was used to assess CD8^+^ T cell proportions. Weighted gene co-expression network analysis was used to identify the co-expression modules with CD8^+^ T cell proportions and bladder tumor purity. Subsequently, we performed correlation analysis among angiogenesis factors, angiogenesis inhibitors, immune inflammatory responses, and CD8^+^ T cell related genes in tumor microenvironment.

**Results:**

A CD8^+^ T cell related co-expression network was identified. Eight co-expressed genes (*PSMB8*, *PSMB9*, *PSMB10*, *PSME2*, *TAP1*, *IRF1*, *FBOX6*, *ETV7*) were identified as CD8^+^ T cell-related genes that promoted infiltration of CD8^+^ T cells, and were enriched in the MHC class I tumor antigen presentation process. The proteins level encoded by these genes (*PSMB10*, *PSMB9*, *PSMB8*, *TAP1*, *IRF1*, and *FBXO6*) were lower in the high clinical grade patients, which suggested the clinical phenotype correlation both in mRNA and protein levels. These factors negatively correlated with angiogenesis factors and positively correlated with angiogenesis inhibitors. PD-1 and PD-L1 positively correlated with these genes which suggested PD-1 expression level positively correlated with the biological process composed by these co-expression genes. In the high expression group of these genes, inflammation and immune response were more intense, and the tumor purity was lower, suggesting that these genes were immune protective factors that improved the prognosis in patients with bladder cancer.

**Conclusion:**

These co-expressed genes promote high levels of infiltration of CD8^+^ T cells in an immunoproteasome process involved in MHC class I molecules. The mechanism might provide new pathways for treatment of patients who are insensitive to PD-1 immunotherapy due to low degrees of CD8^+^ T cell infiltration.

## Introduction

Urothelial carcinomas (UCs) are the fourth most common tumors in developed countries (1). There has been no significant improvement of patient survival over the past 15 years (2). Tumor-related fatality rates for breast cancer, prostate cancer, colorectal cancer, and lung cancer decreased by about 20–40%, while that of bladder cancer decreased by less than 5% (2). For this reason, it is important to identify treatments that improve the prognosis in bladder cancer patients. Bladder cancer is characterized by high mutation rate and many neoplastic antigens (3). Immune checkpoint treatment has become an important treatment method after adjuvant bladder cancer chemotherapy (4). PD-1 is an immune checkpoint protein on T cells that binds to PD-L1 on tumor cells, limits inflammatory and immune responses, and protects tumor cells from T-cell attack (5–8). In recent years, five therapies targeting the programmed cell death protein (PD-1) and programmed cell death ligand 1 (PD-L1) axis were approved for bladder cancer (9), improving prognosis in patients with advanced bladder cancer. Nevertheless, the therapy for progression post PD1/L1 inhibitors is now available but not curative (10). This might be due to the lack of activated T lymphocyte infiltration at the tumor site and the low expression level in the CD8^+^ T lymphocyte (11). These findings suggest that exploring the specific mechanisms of promoting T lymphocytes infiltration may result in improving the effective rate of PD-1 treatment.

CD4^+^ T cells and CD8^+^ T cells are modifiers that determine the clinical response in cancer immunotherapies (12). During antigen processing, exogenous antigen peptides bind to major histocompatibility complex (MHC) class II molecules and modulate immune responses of CD4+ T cells, while endogenous antigen peptides (usually 8–10 amino-acid residues long) bind to major histocompatibility complex (MHC) class I molecules and modulate immune responses of CD8^+^ T cells (13). Tumor antigens are degraded by immunoproteasomes and transporters in antigen-presenting cells (APC) (14), and are recognized by CD8^+^ T cells after binding to MHC class I (15). Bladder cancer patients with high levels of infiltration of CD8^+^ T cells in tumor sites showed better prognosis (16, 17). This suggests that CD8^+^ T cells play an important role in bladder tumor immunity. Weighted gene co-expression network analysis (WGCNA) in the R package identifies co-expressed genes with similar biological functions (18); this algorithm helps identify co-expressed genes that promote CD8^+^ T cell infiltration, and may identify treatment pathways for patients who are not sensitive to immunotherapy because of a low degree of T lymphocyte infiltration.

In this paper, we identified eight co-expression genes promoting CD8^+^ T cells in bladder cancer. These eight genes were involved in MHC class I antigen process, suggesting a positive correlation between MHC class I antigen process and CD8^+^ T cells infiltration level. Next, we explored the correlation of their expression with angiogenic factors, angiogenesis inhibitors, tumor purity, inflammation, and immune responses, and verified correlations of CD8^+^ T cell infiltration in other cancers.

## Materials and Methods

### Data Source

We downloaded The Cancer Genome Atlas (TCGA)-BLCA FPKM data (http://cancergenome.nih.gov/) containing 414 cancer tissue samples and 19 normal tissues. GSE32894 (19) and GSE48075 (20) were also downloaded from the GEO (http://www.ncbi.nlm.nih.gov/geo/) database whose platform is GPL6947. GSE32894 contained 308 urothelial cancer samples and GSE48075 contained 142 primary bladder samples.

### Lymphocyte Proportion and Tumor Purity

CIBERSORT is an algorithm that analyzes the cell proportion in bulk tissue gene expression matrices (21). LM22 is a gene signature matrix that defines 22 immune cell subtypes; it was download from the CIBERSORT website portal (https://cibersort.stanford.edu/). We analyzed CD8^+^ T cell proportions based on the LM22 matrix and CIBERSORT algorithm, and samples with P < 0.05 was considered to be significant and were considered in this study. The Estimation of Stromal and Immune cells in Malignant Tumor tissues using Expression data (ESTIMATE) is a method that infers the fraction of stromal and immune cells using gene expression signatures (22). Using the ESTIMATE package, we calculated stromal Scores, immune Scores, and tumor purity in each bladder cancer sample.

### WGCNA

WGCNA is a system biology approach that converts co-expression correlations into connection weights or topology overlap values (18). We used it to determine CD8^+^ T cell co-expressed genes. The expression patterns were similar for genes involved in the same pathway or biological process (23). In this paper, to build a scale-free topology network, we set the soft threshold as 5, R square = 0.98, and the number of genes in the minimum module as 30. We input the CD8^+^ T cell proportion, stromal scores, immune scores, and tumor purity as phenotype files. In this manner, a cluster of CD8^+^ T cell infiltration-related genes with similar function were identified using WGCNA (24).

### Protein Network and Function Enrichment

The genes were selected using Pearson correlation coefficient >0.4 between genes and CD8^+^ T cell proportions. The co-expression modules of these T cell infiltration-related genes were generated using Cytoscape software. The Database for Annotation, Visualization and Integrated Discovery (DAVID, v6.8) is an open source database that performs function enrichment (25). We used the Kyoto Encyclopedia of Genes and Genomes (KEGG) (https://www.genome.jp/kegg/) (26) and Gene Ontology (GO) (http://geneontology.org/) analysis (27) to identify the biological function in each co-expression module.

### The Human Protein Atlas (HPA)

The HPA database (http://www.proteinatlas.org/) was applied to show the difference of the co-expression genes in protein level, the color intensity was used to assess the protein expression level.

### Immune Microenvironment Correlation Analysis

We explored the correlations between CD8^+^ T cells and angiogenic factors, angiogenesis inhibitors, tumor purity, inflammation, and immune responses. VEGFD (28), PDGFD (29), PDGFRA (30), FGFR1, FGFR2, FGF7, FGF12 (31), TGFBR2, and TGFBR3 (32) were considered angiogenic factors. IL12A, IL12B, IL12RB1, IL12RB2, IL10RA, IFNL1, IFNL2, and IFNL3 (33) were considered angiogenic inhibitors. Seven metagene sets included lymphocyte‐specific kinase (LCK), hemopoietic cell kinase (MCK), major histocompatibility complex class I (MHC‐I), immunoglobulin G (IgG), major histocompatibility complex class II (MHC II), signal transducer and activator of transcription 1 (STAT1), and interferon (34). All of these were considered different types of inflammation and immune responses. Next, we calculated the correlations between CD8^+^ T cell infiltration genes and tumor purity based on TCGA.

### GSEA

Gene set enrichment analysis (GSEA) is a calculation method that determines the significance and consistency differences of a predefined dataset between two biological states (35). The gene matrix in TCGA was divided into high and low expression groups, in accordance with the median expression level of CD8^+^ T cell infiltration-related genes. Based on allocation, biological functions related to the high expression group was identified, allowing us to identify the mechanisms underlying the role of CD8^+^ T cell infiltration-related co-expression genes.

### Pan-Cancer Analysis

The Tumor Immune Estimation Resource (TIMER; https://cistrome.shinyapps.io/timer/) (36) was used to analyze the correlations between CD8^+^ T cells and 33 types of cancer. A correlation coefficient >0.4 was considered significant.

### Statistical Analysis

Statistical analysis was carried out using GraphPad Prism 8 and R 3.6.3 (https://www.r-project.org/). Student’s t-tests are used to analyze expression differences and CD8^+^ T cell proportion differences in subgroups. Co-expression coefficients were calculated using the Pearson correlation. The subgroups were divided based on the median value. Kaplan–Meier survival analysis was applied to generate overall survival curves and the log-rank test was used to calculate the significance. Independent prognostic factors were selected using the univariate Cox regression method. The “survival,” “ggplot2,” “corrplot,” “pheatmap,” and “limma” packages were built using R version 3.6.3. Differences with *P* < 0.05 were significant.

## Results

### CD8^+^ T Cell Related Modules

**Figure 1:**
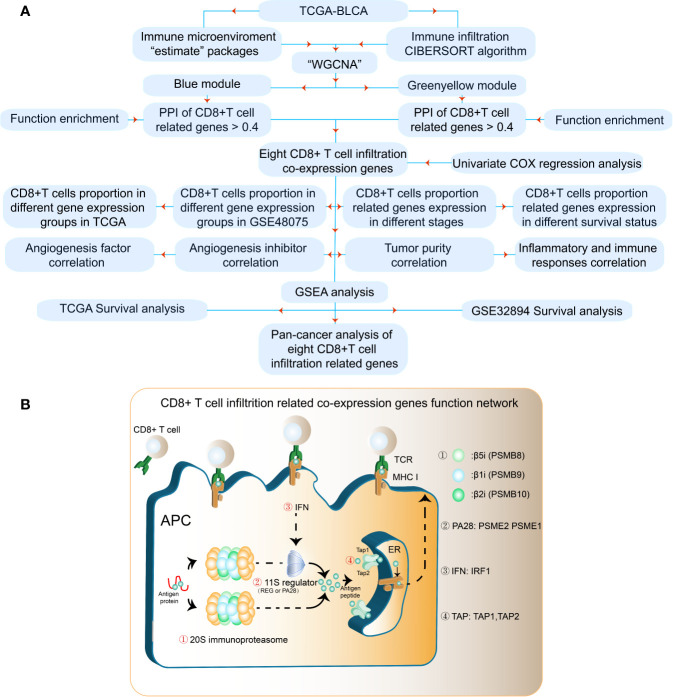
Flowchart for identifying CD8^+^ T cell-promoting co-expressed genes. **(A)** TCGA-BLCA FPKM contained 414 cancer tissue samples and 19 normal tissues. GSE32894 contained 308 urothelial cancer samples and GSE48075 contained 142 primary bladder samples. WGCNA was used generate a co-expression network. GO analyses were applied to identify CD8^+^ T cell-related modules. Independent prognostic factors were selected using univariate Cox regression. **(B)** The antigen peptide presentation process is shown. Immunoproteasomes are composed of 20S subunits. PSMB8, PSMB9, and PSMB10 are the core of the 20S subunit. PA28 (PSEM2) is a regulator of immunoproteasomes that enhances the activity of the 20S subunit. Transporters associated with antigen processing (TAP1 and TAP2) reside in the endoplasmic reticulum (ER), and transport antigen peptides into the ER. The IFN-regulatory factor 1 protein (IRF1) is up-regulated by IFNγ, and upregulates MHC class I antigen peptide presentation-related processes.

The results of our methodology are explained in **Figure 1A**. The interactions among CD8^+^ T cell infiltration-related co-expressed genes are shown in **Figure 1B**.

**Figure 2:**
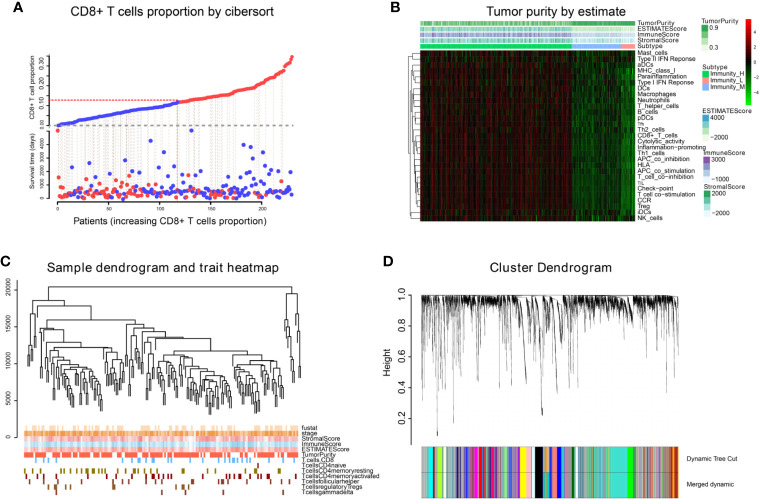
WGCNA analysis. **(A)** The infiltration proportion of CD8+ T cells in TCGA. **(B)** The immune score, estimate score, stromal score, and tumor purity of TCGA-BLCA are shown, and were inputted as phenotype information for WGCNA analysis. **(C)** We clustered the samples by cut Height = 20,000, and 226 samples were included. The sample dendrogram of 226 samples and trait map are shown. **(D)** Hierarchical clustering tree was built using the dynamic hybrid cutting method, where each leaf on the tree represents a gene, and each branch represents a co-expression module; 28 co-expression models were generated.

**Figure 3:**
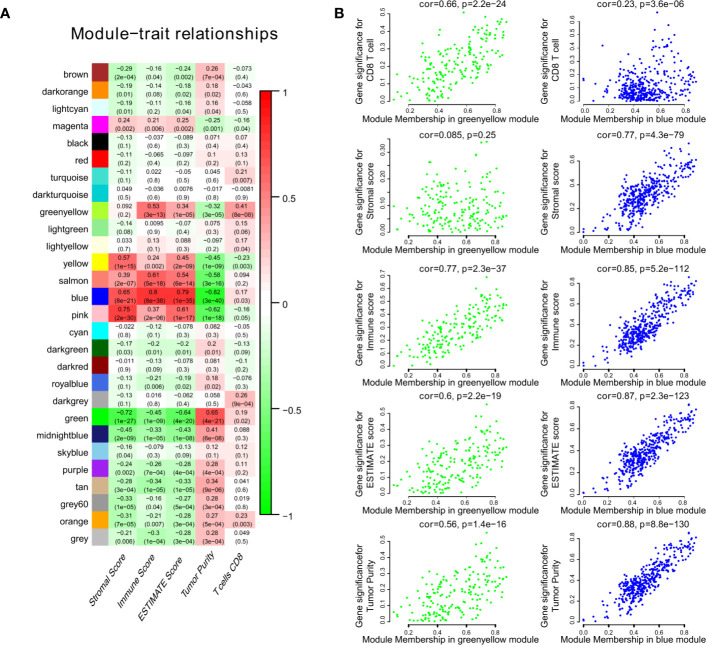
The result of WGCNA. **(A)** The correlation coefficient between different phenotypes and co-expression modules were showed. **(B)** The blue module was highly correlated to stromal score (R^2^ = 0.77, P = 4.3e^-79^), immune score (R^2^ = 0.85, P = 5.2e^-112^), estimate score (R^2^ = 0.87, P = 2.3e^-123^), and tumor purity (R^2^ = 0.88, P = 8.8e^-130^), while the green-yellow module showed higher correlation with CD8^+^ T cells (R^2^ = 0.66, P = 2.2e^-24^).

**Figure 4:**
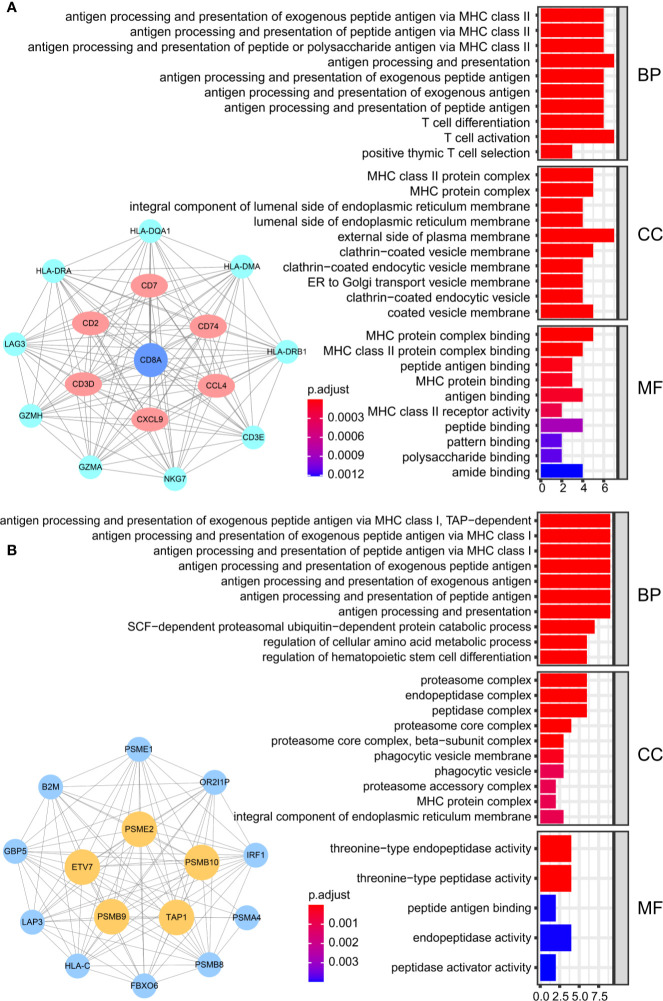
The result of GO analysis. **(A)** The genes in the blue module were enriched in antigen processing and presentation *via* MHC class II molecules. **(B)** The genes in green-yellow module were enriched in antigen processing and presentation *via* MHC class I molecules.

**Figure 5:**
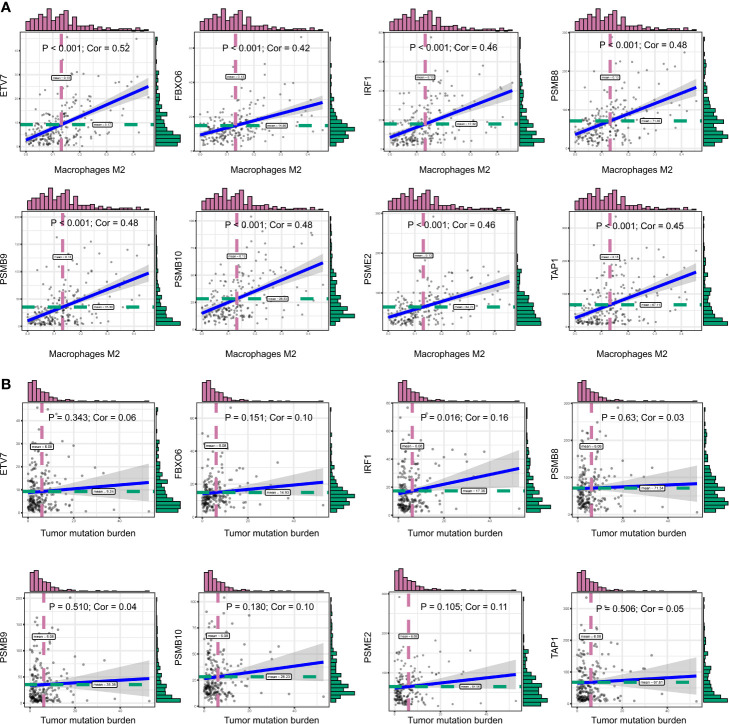
**(A)** The correlation between co-expression genes and CD8+ T cells proportion. **(B)** The correlation between co-expression genes and tumor mutation burden (TMB).

We obtained 243 samples with complete clinical information and proportion of immune cell infiltration assessment. The proportion of CD8+ T cells and the survival status are illustrated in **Figure 2A**, the red point means death status at the end point. The tumor purity heatmap is illustrated in **Figure 2B**. We clustered the samples by cut Height = 20,000, and 226 samples were included. The sample dendrogram of 226 samples and trait map are illustrated in **Figure 2C**. To build a co-expression network, we used a dynamic hybrid cutting method to build a hierarchical clustering tree, where each leaf on the tree represents a gene, and each branch represents a co-expression module; 28 co-expression models were generated (**Figure 2D**). The correlation coefficients among various phenotypes and co-expression modules were calculated (**Figure 3A**); 32 CD8^+^ T cell infiltration-related co-expressed genes were selected with correlation coefficients >0.4. The gene significances for these 32 CD8^+^ T cells related genes are displayed in **Table 1**. The 32 genes were mostly involved in the blue and green-yellow modules. The blue module was highly correlated with stromal Score (R^2^ = 0.77, P = 4.3e^-79^), immune score (R^2^ = 0.85, P = 5.2 e^-112^), estimate score (R^2^ = 0.87, P = 2.3e^-123^), and tumor purity (R^2^ = 0.88, P = 8.8e^-130^), while the green-yellow module showed higher correlation with CD8^+^ T cells (R^2^ = 0.66, P = 2.2e^-24^) (**Figure 3B**). Next, we explored the functions of the blue and green-yellow modules. The genes in the blue module were enriched in antigen process and presentation *via* MHC class II molecules (**Figure 4A**), while the genes in the green-yellow module were enriched in antigen process and presentation *via* MHC class I molecules (**Figure 4B**). The univariate Cox regression method was used to calculate the independent prognostic effect of 32 genes (**Table 2**). We focused on the green-yellow module based on the antigen presentation *via* MHC class I molecules, and we were interested in several genes (*PSMB8*, *PSMB9*, *PSMB10*, *PSME2*, *TAP1*, *IRF1*, *FBOX6*, *ETV7*) with independent prognostic effects (*p* < 0.01). The correlation between co-expression genes and CD8+ T cells proportion was showed in **Figure 5A**. The correlation between co-expression genes and tumor mutation burden (TMB) was showed in **Figure 5B**, although the correlation was not statistically significant, positive correlation between co-expression genes and tumor mutation burden was shown.

**Table 1:** The Module and Gene significance for CD8^+^ T Cells related genes.

ID	Module	GS.T.cells.CD8^+^	P-Value
CD8A	blue	0.668	1.95E-22
NKG7	blue	0.572	1.44E-15
GZMH	blue	0.537	1.40E-13
PSME1	green yellow	0.508	4.54E-12
CCL4	blue	0.499	1.23E-11
PSME2	green yellow	0.483	6.74E-11
ETV7	green yellow	0.470	2.50E-10
LAG3	blue	0.469	2.69E-10
GZMA	blue	0.462	5.35E-10
PSMB9	green yellow	0.456	9.61E-10
TAP1	green yellow	0.453	1.23E-09
CXCL9	blue	0.452	1.34E-09
IRF1	green yellow	0.452	1.35E-09
CD2	blue	0.443	3.27E-09
PSMB8	green yellow	0.441	3.81E-09
GBP5	green yellow	0.434	7.03E-09
OR2I1P	green yellow	0.426	1.46E-08
CD3D	blue	0.423	1.87E-08
CTSW	blue	0.418	2.74E-08
B2M	green yellow	0.418	2.79E-08
CD74	blue	0.414	3.92E-08
LAP3	Green yellow	0.414	3.96E-08
HLA-DRB1	blue	0.412	4.75E-08
CD7	blue	0.412	4.80E-08
HLA-DRA	blue	0.411	5.00E-08
PSMA4	green yellow	0.409	5.95E-08
CD3E	blue	0.408	6.27E-08
FBXO6	green yellow	0.408	6.46E-08
PSMB10	green yellow	0.408	6.57E-08
HLA-C	green yellow	0.406	7.68E-08
HLA-DMA	blue	0.405	8.43E-08
HLA-DQA1	blue	0.403	9.76E-08

GS, Gene significance.

**Table 2:** Univariate Cox proportional hazard analysis of CD8^+^ T cells related genes.

ID	HR	HR.95L	HR.95H	P-value
PSMB10	0.980	0.968	0.991	0.0005
FBXO6	0.958	0.934	0.982	0.0007
PSMB8	0.994	0.990	0.998	0.002
ETV7	0.959	0.934	0.985	0.0024
IRF1	0.978	0.963	0.993	0.0041
TAP1	0.995	0.991	0.998	0.0052
PSME2	0.993	0.988	0.998	0.0087
PSMB9	0.992	0.986	0.998	0.0098
CD7	0.953	0.917	0.989	0.0120
B2M	0.999	0.999	0.999	0.0128
HLA-C	0.999	0.999	0.999	0.0148
CD74	0.999	0.999	0.999	0.0161
OR2I1P	0.983	0.969	0.997	0.0165
CD3D	0.977	0.959	0.996	0.0188
LAG3	0.956	0.920	0.993	0.0223
PSMA4	0.977	0.958	0.997	0.0232
HLA-DRA	0.999	0.999	0.999	0.0234
PSME1	0.996	0.994	0.999	0.0234
CD2	0.976	0.956	0.997	0.0242
HLA-DMA	0.994	0.988	0.999	0.0244
NKG7	0.987	0.975	0.999	0.0318
LAP3	0.992	0.984	0.999	0.0423
CCL4	0.968	0.938	0.999	0.0445
CD3E	0.974	0.948	0.999	0.0445
GBP5	0.983	0.966	0.999	0.0470
CD8A	0.964	0.927	1.002	0.0634
HLA-DQA1	0.993	0.985	1.000	0.0743
GZMA	1.001	0.999	1.003	0.0753
HLA-DRB1	0.999	0.999	1.000	0.0774
GZMH	0.978	0.953	1.005	0.1040
CTSW	0.985	0.965	1.005	0.1313
CXCL9	0.997	0.993	1.0012	0.1780

HR, hazard ratio.

### Clinical Phenotype Analysis

**Figure 6:**
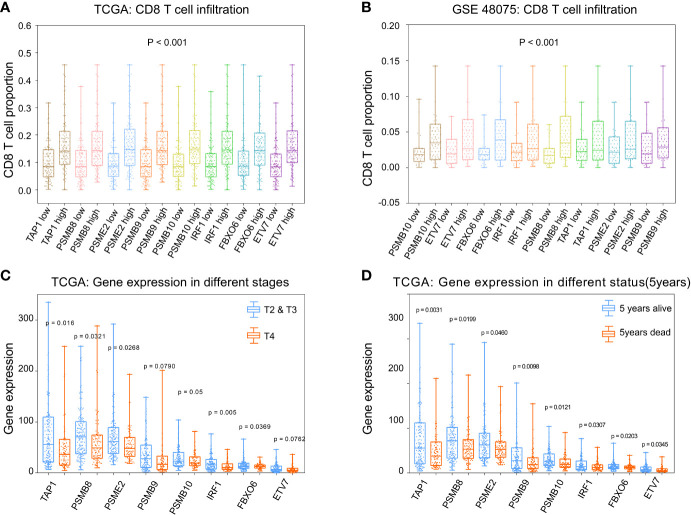
Phenotype analysis of *PSMB8*, *PSMB9*, *PSMB10*, *PSME2*, *TAP1*, *IRF1*, FBOX6, and *ETV7* with CD8^+^ T cell infiltration proportions. **(A)** There was higher infiltration proportion in high expression groups (*p* < 0.05) of *PSMB8*, *PSMB9*, *PSMB10*, *PSME2*, *TAP1*, *IRF1*, *FBOX6*, and ETV7, suggesting that these genes promote CD8^+^ T cell infiltration in TCGA. **(B)** Higher infiltration proportions in high expression groups of *PSMB8*, *PSMB9*, *PSMB10*, *PSME2*, *TAP1*, *IRF1*, *FBOX6*, and *ETV7*, suggesting that these genes promote CD8^+^ T cell infiltration in GSE48075. **(C)** Expression level in stages 2/3 of these genes were higher than in stage 4. **(D)** Expression levels in the 5-year survival group of these genes were higher than those of the 5-year death group.

To calculate the correlations between these and CD8^+^ T cell infiltration proportions, subgroups were created according to the median of eight gene expression values in the TCGA-BLCA (**Figure 6A**) and GSE48075 (**Figure 6B**) cohorts. We found higher infiltration proportions in high expression groups (*p* < 0.05), suggesting that these genes promote CD8^+^ T cell infiltration. CD8^+^ T cell infiltration improves prognosis. Then, the various stages and statuses were applied to determine the prognosis level. For these eight genes, expression levels in stage 4 (**Figure 6C**) and 5-year mortality (**Figure 6D**) groups were significantly lower (p < 0.05).

### Immune Microenvironment Analysis

**Figure 7:**
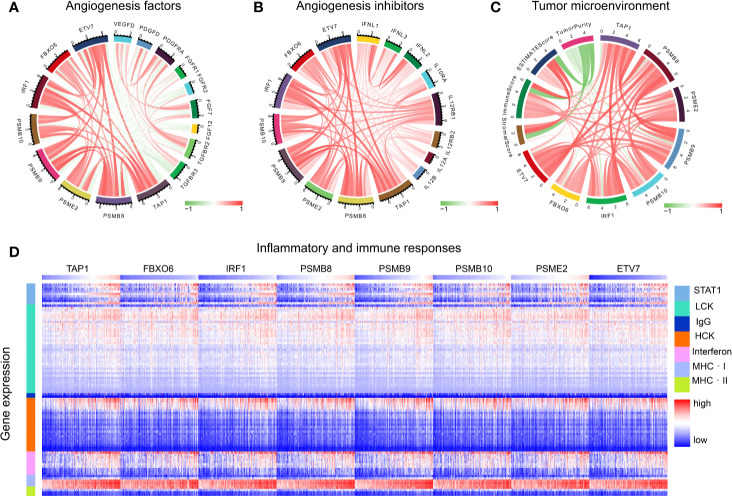
Tumor microenvironment analysis. **(A)** The correlation of CD8+ T cells infiltration promoting genes to angiogenesis factors. **(B)** The correlation of CD8+ T cells infiltration promoting genes to angiogenesis inhibitors. **(C)** The correlation of CD8^+^ T cell infiltration-promoting genes to tumor purity. **(D)** The correlation of CD8^+^ T cell infiltration-promoting genes to inflammatory and immune responses. These responses were induced by lymphocyte‐specific kinase (LCK), hemopoietic cell kinase (MCK), major histocompatibility complex class I (MHC‐I), immunoglobulin G (IgG), major histocompatibility complex class II (MHC II), signal transducer, activator of transcription 1 (STAT1), and interferon.

The functions of angiogenic factors and angiogenesis inhibitors are listed in **Table 3**. A negative correlation was found between several genes (*PSMB8*, *PSMB9*, *PSMB10*, *PSME2*, *TAP1*, *IRF1*, *FBOX6*, *ETV7*) and angiogenic factors (**Figure 7A**); positive correlations were found for angiogenic inhibitors (**Figure 7B**), suggesting that these genes might modulate vascular changes in the tumor microenvironment. With the increase of CD8^+^ T cell infiltration-related gene expression, there was a decreasing trend of tumor purity (**Figure 7C**). These findings suggest that these genes might influence bladder tumor purity and local microenvironment component proportions. To analyze the correlation between the eight genes and immune responses, we choose seven metagenes representing various types of inflammatory and immune responses. We found that *PSMB8*, *PSMB9*, *PSMB10*, *PSME2*, *TAP1*, *IRF1*, *FBOX6*, and *ETV7* positively associated with six of these clusters, except IgG (**Figure 7D**).

**Table 3:** The list of angiogenic factors and inhibitors.

ID	Function	Name
VEGFD	Lymphangiogenic growth factor	Vascular endothelial growth factor D
PDGFD	A specific, protease-activated ligand for the PDGF beta-receptor.	Platelet-derived growth factor D
PDGFRA	Recruit smooth muscle cells	Platelet-derived growth factor receptor alpha
FGFR1, FGFR2	Stimulate angio/arteriogenesis	Fibroblast growth factor receptor1/2
FGF7, FGF12	Stimulate angio/arteriogenesis	Fibroblast growth factor 7/12
TGFBR2, TGFBR3	Stimulate extracellular matrix production	Type II/III TGF-beta receptor
IL12A, IL12B	Inhibit endothelial migration. Downregulate bFGF. Induce interferon-g and IP-10	Interleukin-12 subunit alpha/beta
IL12RB1, IL12RB2	Inhibit endothelial migration. Downregulate bFGF. Induce interferon-g and IP-10	Interleukin-12 receptor beta1/2
IL10RA	Inhibit endothelial migration	Interleukin 10 receptor subunit alpha
IFNL1, IFNL2, IFNL3	Inhibit endothelial migration; Downregulate bFGF	Interferon lambda 1/2/3

### GSEA Analysis and Survival Analysis

**Figure 8:**
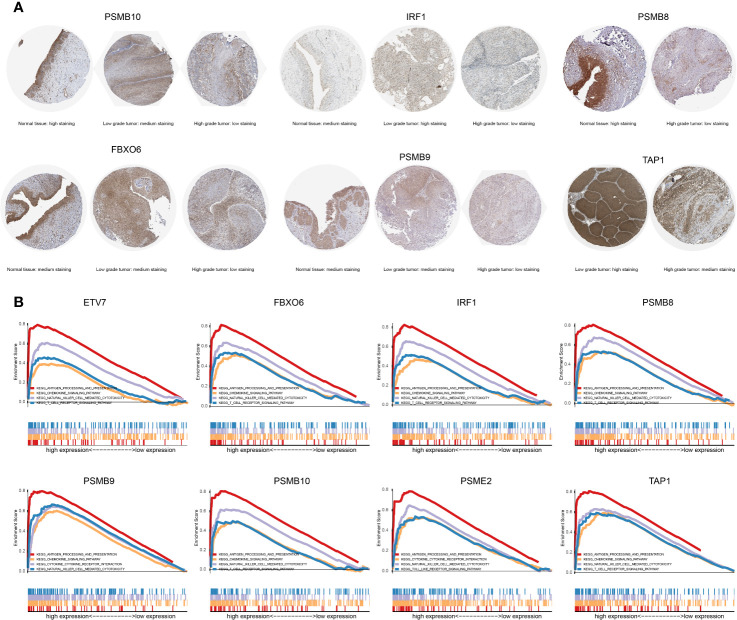
**(A)** The proteins level encoded by these genes (*PSMB10*, *PSMB9*, *PSMB8*, *TAP1*, *IRF1*, and *FBXO6*) were lower in the high clinical grade patients in the human protein atlas (HPA). **(B)** The results of GSEA analysis. Antigen processing and presentation, the chemokine signaling pathway, nature killer cell-mediated cytotoxicity, and the T cell receptor signaling pathway were related to the high expression group in *ETV7*, *FBXO6*, *IRF1*, *PSMB8*, *PSMB9*, *PSMB10*, *PSME2*, and *TAP1*.

**Figure 9:**
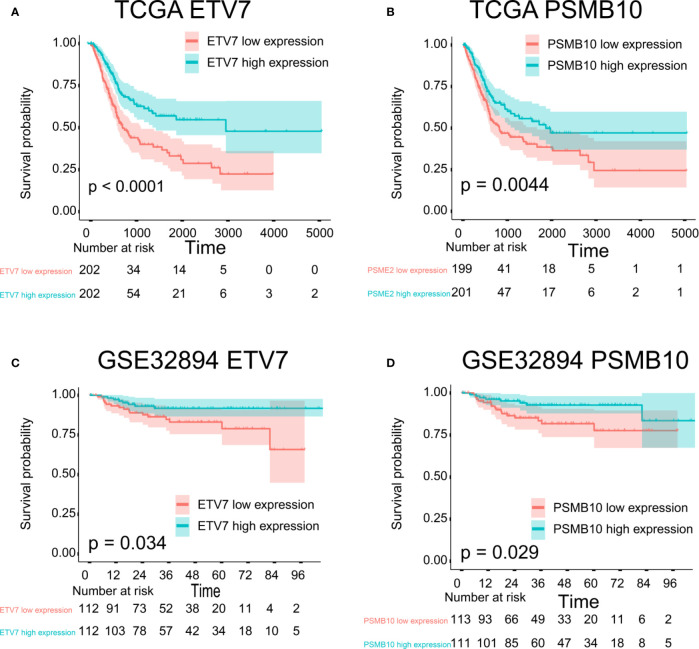
Survival analysis. **(A)** ETV7 survival analysis in TCGA, *P* < 0.0001. **(B)** PSMB10 survival analysis in TCGA, *P* = 0.0044. **(C)** ETV7 survival analysis in GSE32894, *P* = 0.034. **(D)** PSMB10 survival analysis in GSE32894, *P* = 0.029.

The proteins level encoded by these genes (*PSMB10*, *PSMB9*, *PSMB8*, *TAP1*, *IRF1*, and *FBXO6*) were lower in the high clinical grade patients in the human protein atlas (HPA), which suggested the clinical phenotype correlation both in mRNA and protein levels (**Figure 8A**). GSEA analysis showed that antigen processing and presentation, chemokine signaling pathway, nature killer cell mediated cytotoxicity, and the T cell receptor signaling pathway were related to the high expression group (**Figure 8B**). The P-value is displayed **Table 4**. We found that these biological pathways were immune-related and were involved in tumor immunity that protects against tumor infiltration. To analyze their influence on overall survival, we performed survival analysis. The patients in low expression groups for *PSMB10* (TCGA: *P* = 0.0044; GSE32894: *P* = 0.029) and *ETV7* (TCGA: *P* < 0.0001; GSE32894: *P* = 0.034) showed survival risk against high expression groups (**Figure 9**). Despite the fact that no significant difference was detected for *PSMB8*, *PSMB9*, *PSME2*, *TAP1*, *IRF1*, or *FBOX6*, these patients showed more survival risk trends in low expression groups.

**Table 4:** The results of GSEA analysis.

ID	Antigen processing and presentation		Chemokine signaling pathway		Nature killer cell mediated cytotoxicity		T cell receptor signaling pathway	
	NOM-p	FDR-q	NOM-p	FDR-q	NOM-p	FDR-q	NOM-p	FDR-q
PSMB8	<0.0001	<0.0001	<0.0001	0.0037	<0.0001	<0.0001	0.0020	0.0082
PSMB9	<0.0001	0.0002	<0.0001	0.0002	<0.0001	<0.0001	0.0020	0.0020
PSMB10	<0.0001	<0.0001	0.0080	0.0080	<0.0001	<0.0001	0.0158	0.0150
PSME2	<0.0001	<0.0001	0.0368	0.1149	<0.0001	<0.0001	0.06	0.1486
TAP1	<0.0001	<0.0001	<0.0001	<0.0001	<0.0001	<0.0001	<0.0001	0.0008
IRF1	<0.0001	<0.0001	0.0110	0.0272	<0.0001	<0.0001	0.0056	0.0133
ETV7	<0.0001	<0.0001	0.0019	0.0143	<0.0001	<0.0001	0.0320	0.0729
FBXO6	<0.0001	<0.0001	<0.0001	0.0015	<0.0001	<0.0001	0.0060	0.0066

### Pan-Cancer Analysis

**Figure 10:**
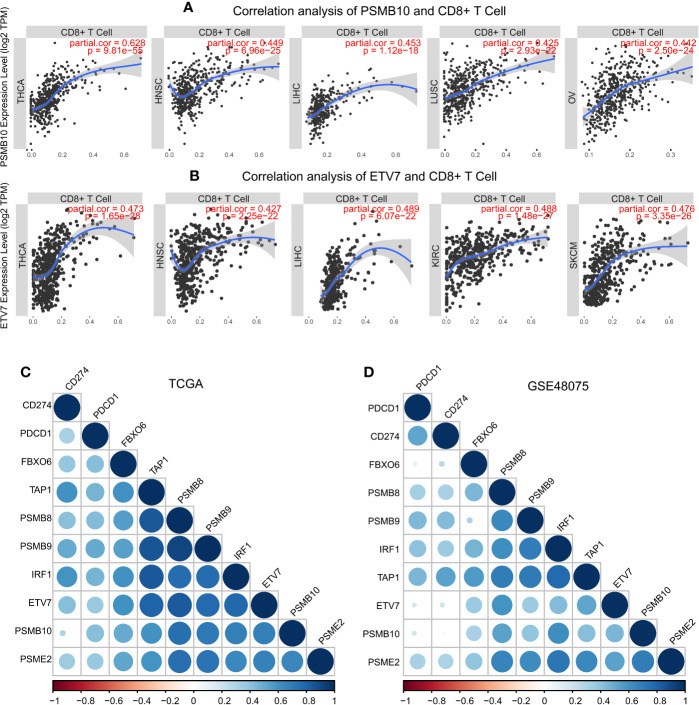
Pan-cancer analysis of PSMB10 and ETV7. **(A)** Correlation analysis of PSMB10 and CD8^+^ T cells. **(B)** Correlation analysis of ETV7 and CD8^+^ T cells. **(C, D)** PD1/PDL1 correlation analysis. A positive correlation was found between CD8^+^ T cells and PD1/PDL1 in TCGA and GSE48075.

These results demonstrated the role of *PSMB8*, *PSMB9*, *PSMB10*, *PSME2*, *TAP1*, *IRF*, *FBOX6*, and *ETV7* in bladder cancer. Next, we analyzed the correlation between these genes and CD8^+^ T cell infiltration in other types of cancers. *IRF1*, *PSMB9*, *TAP1*, *ETV7*, and *PSMB10* were related to CD8^+^ T cell infiltration proportion in thyroid carcinoma, breast invasive carcinoma, head and neck squamous cell carcinoma, hepatocellular carcinoma, lung adenocarcinoma, and skin cutaneous melanoma (**Table 5**). The correlations between *ETV7* and *PSMB10* and CD8^+^ T cell infiltration in other types of cancer are shown in **Figure 10A**.

**Table 5:** Correlation Analysis of Candidate Factors and CD8^+^ T cells.

Gene	Count	Cancer type (CD8^+^ T cells cor > 0.4)
IRF1	13	ACC, BRCA, CESC, HNSC, KIRC, KIRP, LIHC, LUAD, LUSC, PAAD, PRAD, STAD, SKCM
PSMB9	10	THCA, BRCA-Her2, HNSC - HPVpos, KIRC, LIHC, LUAD, LUSC, OV, STAD, SKCM
TAP1	9	THCA, BRCA-Her2, CESC, KIRC, LIHC, LUAD, LUSC, UCS, SKCM
ETV7	7	THCA, BRCA-Her2, CHOL, HNSC, KIRC, LIHC, SKCM,
PSMB10	6	THCA, DLBC, HNSC, LIHC, LUSC, OV,
PSMB8	4	THCA, KIRC, LUSC, SKCM,
PSEM2	2	THCA, SKCM
FBOX6	1	THCA

ACC, Adrenocortical carcinoma; BRCA, Breast invasive carcinoma; CESC, Cervical squamous cell carcinoma and endocervical adenocarcinoma; CHOL, Cholangiocarcinoma; DLBC, Lymphoid Neoplasm Diffuse Large B-cell Lymphoma; HNSC, Head and Neck squamous cell carcinoma; KIRC, Kidney renal clear cell carcinoma; KIRP, Kidney renal papillary cell carcinoma; LIHC, Liver hepatocellular carcinoma; LUAD, Lung adenocarcinoma; LUSC, Lung squamous cell carcinoma; OV, Ovarian serous cystadenocarcinoma; PAAD, Pancreatic adenocarcinoma; PRAD, Prostate adenocarcinoma; SKCM, Skin Cutaneous Melanoma; STAD, Stomach adenocarcinoma; THCA, Thyroid carcinoma; UCS, Uterine Carcinosarcoma.

## Discussion

There is a growing body of evidence to suggest that anti-PD-1 therapy (primary resistance) is not effective in most bladder patients. This might be due to the lack of activated T lymphocyte infiltration at the tumor site (10, 11). In tumor immunity, tumor antigen peptides bind to MHC class I molecules, and mediate cellular immune. In the present study, we attempted to identify co-expression genes that promote CD8^+^ T cell infiltration based on the WGCNA algorithm. This method identified cluster of co-expressed genes promoting CD8^+^ T cell infiltration with the same biological function. The identification of the function of these factors may help uncover the process of promoting CD8^+^ T cell infiltration and to identify candidate correlation factors. We identified two CD8^+^ T cell co-expression modules. To select the module with immune-related function, we performed function enrichment. The genes in green-yellow modules were mostly involved in MHC class I process and presentation and proteasome in antigen presenting cells. The genes in the blue module were involved in MHC class II process and presentation. Therefore, we focused on the genes in the green-yellow module with CD8^+^ T cell proportion correlation >0.4. *PSMB8*, *PSMB9*, *PSMB10*, *PSME2*, *TAP1*, *IRF1*, *FBOX6*, and *ETV7* were identified as CD8^+^ T cell infiltration-promoting factors with independent prognostic effects.

In the early 1990s, proteasome 20S subunit beta 8 (PSMB8, also known as LMP7) and proteasome 20S subunit beta 9 (PSMB9, also known as LMP2) were identified as proteasome subunits β5i and β1i (37–40). β5i and β1i are highly homologous to β5 and β1, which are the major components of the 20S proteasome (41). Another proteasome β2 homologous subunit β2i was identified that was encoded by proteasome 20S subunit beta 10 (PSMB10) (42–44). The proteasome has a 26S structure, including a 20S central unit and a 19S regulator. After IFN or TNF stimulation, expression levels of these three antigens process related subunits (β5i, β1i, and β2i) are upregulated, and there is neosynthesis of 20S proteasomes were called 20S immunoproteasomes (45, 46). The function of immunoproteasome is different from that of the constitutive proteasome. The antigen peptide which combined with MHC class I, hydrolyzed by immunoproteasome, has stronger CTL activation effect than that in constitutive proteasome (41, 47, 48). Gabriela et al. demonstrated a negatively impact on MHC-I surface expression and antigen presentation process in immunoproteasome triple knockout mice, and cytotoxic activity of CD8^+^ T cell showed a consequently reduced tendency (49). Nagayama et al. suggested that Th1 235 and Th17 differentiation was inhibited by a selective inhibitor of immunoproteasome in the murine model (50). Cathro et al. found a significant difference of immunoproteasome components in different urothelium carcinoma stages, which the immunoproteasome components in low stage level is higher (51). These findings suggest that *PSMB8*, *PSMB9*, and *PSMB10* might promote CD8^+^ T cell infiltration based on expression of greater numbers of immunoproteasome 20S core units in bladder cancers.

The immunoproteasome is regulated by the 11S regulator (known as REG or PA28) which enhances the activity of peptidase and stimulates the production of antigen peptide (52). Proteasome activator 28 (PA28) is composed of PA28α and PA28β subunits encoded by *PSME1* and *PSME2* (53, 54). PA28 enhances the MHC class I antigen process by influencing peptide cleavage and release (55, 56). PA28β subunits might induce PA28α complex formation, thereby enhancing immunoproteasome activity in antigen presenting cells (57). These findings suggest that PA28β improves the MHC class I process, and the induces more effector T cells by increasing antigen presentation.

Transporters associated with antigen processing (*TAP1* and *TAP2*) reside in the endoplasmic reticulum (ER) and transport antigen peptides into the ER, which plays a crucial role in the MHC class I antigen process (58, 59). Endoplasmic reticulum without *TAP1* and *TAP2* induces the dysfunction of class I MHC molecules (60–62), thereby inhibiting the activity of CD8^+^ T cells. TAP was demonstrated have a prognostic protective effect in lung cancer, cervical cancer, breast cancer, and head and neck cancer (63–66).

The IFN-regulatory factor 1 protein (IRF1) is up-regulated by IFNγ (67). IFNγ upregulates MHC class I antigen peptide presentation-related processes, and enhances the activity of immunoproteasome subunits (*PSMB8*, *PSMB9*, *PSMB10*), regulators (*PSME1*, *PSME2*), and transporters associated with antigen processing (*TAP1*, *TAP2*) (68). Leigh et al. (67) found a paucity of CD8^+^ T Cells in mice, induced by the lack of *TAP1* and *PSMB9* regulated by *IRF1*. These findings suggest that *PSMB8*, *PSMB9*, *PSMB10*, *PSME2*, *TAP1*, and *IRF1* may increase the CD8^+^ T cell proportions by enhancing MHC class I antigen processing and presentation.

In our study, *ETV7* and *FBXO6* were found to be co-expressed with *PSMB8*, *PSMB9*, *PSMB10*, *PSME2*, *TAP1*, and *IRF1*. ETS variant transcription factor 7 (ETV7) is a member of the ETS transcription factors family, which are involved in cellular development and differentiation (69). F-box protein 6 (FBXO6) is a subunit of the ubiquitin protein ligase complex (70). Previous studies have demonstrated that FBXO6 inhibits tumor invasion in gastric and lung cancer; however, the underling mechanisms were not clear (71). In our co-expression analysis, we found that FBXO6 and ETVT were co-expressed with immunoproteasomes 20S, interferon-gamma regulator IRF-1, and protease activator PA28. These findings suggest that there may be a previously undiscovered pathway regulation between these two factors and the MHC I antigen presentation process.

Mariathasan et al. (72) found that the combination of TGF inhibitor- blocking and anti-pd-l1 antibody attenuated the signal transduction of TGF in stromal cells, promoted the chemotaxis of T lymphocyte cells to the tumor center, stimulated strong anti-tumor immunity effect in the mouse model. We determined the role of these factors in the clinical phenotype and tumor microenvironment, we were surprised to find that, when these co-expression factors were highly expressed, the purity of the tumor was significantly reduced, the expression of TGFBR2 and the TGFBR3 were declined, the immune inflammatory response was weakened, the clinical stage of the patient was reduced, and the 5-year survival prognosis improved. Angiogenic factors also play an important role in tumor progression.

We also found that, when expression levels of these factors are low, expression levels of angiogenic factors increase, which lead poor prognosis. We believe that these phenotypic changes are caused by these co-expression factors that enhance the process of synthesis, degradation, and transmission of tumor antigen peptides in antigen-presenting cells, thereby increasing the activity of CD8^+^ T cells.

Infiltration of CD8^+^ T cells is a precondition for tumor immunity in the tumor microenvironment (73). The adaptive immune response was enhanced as antigen recognition increased (74). There are currently many mechanisms that can cause anti-PD1 drug resistance, including the decline in CD8^+^ T cell infiltration, antigen recognition disorders, and defective PD-1 expression. A correlation analysis is shown in **Figure 10** that demonstrates that these genes promote PD-1 expression. These data suggest that the *PSMB8*, *PSMB9*, *PSMB10*, *PSME2*, *TAP1*, *IRF1*, *FBOX6*, and *ETV7* co-expression network might improve anti-PD1 drug resistance by these mechanisms.

This article had some shortcomings. The first point was that only three cohort samples were included in this paper, more cohorts are needed for cross-validation. The second point was that this article only discussed the differences in mRNA and protein levels initially, the mechanisms still needed to be further explored.

In conclusion, *PSMB8*, *PSMB9*, *PSMB10*, *PSME2*, *TAP1*, *IRF1*, *FBOX6*, and *ETV7* are CD8^+^ T cell infiltration-promoting factors. *PSMB8*, *PSMB9*, *PSMB10*, *PSME2*, *TAP1*, and *IRF1* promote CD8^+^ T cell infiltration by enhancing MHC class I tumor antigen processing. Nevertheless, the mechanisms of action of *FBOX6* and *ETV7* remain unclear. This work might generate new concepts for development of anti-PD1 therapy for insensitive bladder patients, and may improve the prognosis in advanced bladder cancer.

## Data Availability Statement

Publicly available datasets were analyzed in this study. This data can be found here: The datasets TCGA-BLCA for this study can be found in the [The Cancer Genome Atlas] [http://cancergenome.nih.gov/)].The datasets GSE32894 and GSE48075 for this study can be found in the [GEO] [http://www.ncbi.nlm.nih.gov/geo/].

## Author Contributions

YW conceived and designed the experiments, authored or reviewed drafts of the paper, and approved the final draft. KY analyzed the data, prepared figures and/or tables, and approved the final draft. JL performed the experiments, authored or reviewed drafts of the paper, and approved the final draft. YL performed the experiments, authored or reviewed drafts of the paper, and approved the final draft. JW performed the experiments, authored or reviewed drafts of the paper, and approved the final draft. XJL performed the experiments, authored or reviewed drafts of the paper, and approved the final draft. XXL performed the experiments, authored or reviewed drafts of the paper, and approved the final draft. ZH performed the experiments, authored or reviewed drafts of the paper, and approved the final draft. JS performed the experiments, authored or reviewed drafts of the paper, and approved the final draft. SS performed the experiments, authored or reviewed drafts of the paper, and approved the final draft. JB conceived and designed the experiments, authored or reviewed drafts of the paper, and approved the final draft. All authors contributed to the article and approved the submitted version.

## Conflict of Interest

The authors declare that the research was conducted in the absence of any commercial or financial relationships that could be construed as a potential conflict of interest.
